# Supplementary material for: Endothelial LRP1 regulates metabolic responses by acting as a co-activator of PPARγ
Source: Nat Commun. 2017 Apr 10;8:14960. doi: 10.1038/ncomms14960 (PMC5394236; doi:10.1038/ncomms14960)
Supplement: Supplementary Information — Supplementary figures, supplementary table and supplementary references. [file ncomms14960-s1.pdf]

SUPPLEMENTARY INFORMATION

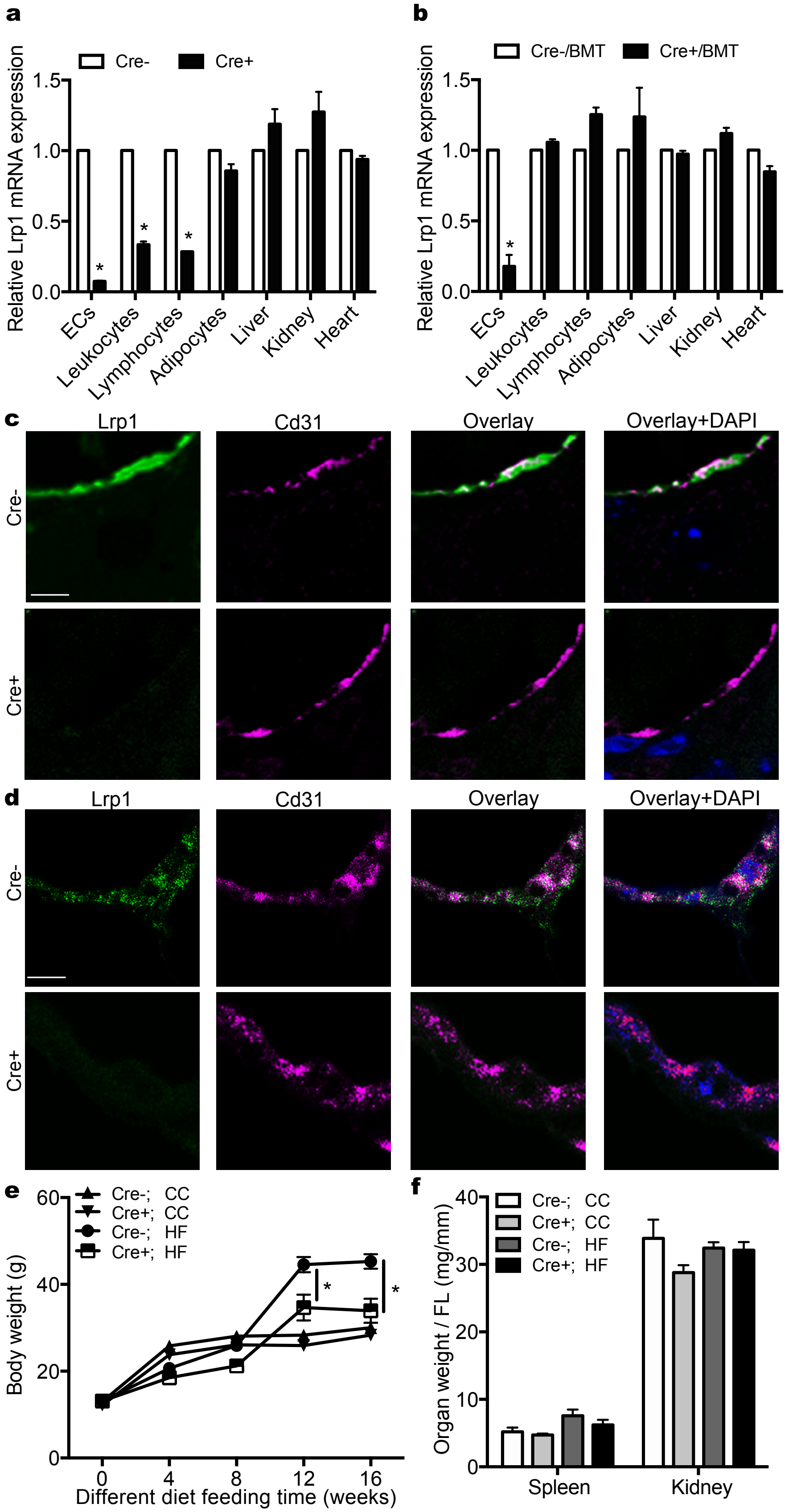

Supplementary Figure 1 Mice with Tie2Cre-mediated Lrp1 depletion display decreased body weight gain. (**a**) Mice with Tie2Cre mediated Lrp1 depletion ($Lrp1^{f/f}$;$Tie2Cre^{+}$; Cre+) displayed decreased Lrp1 expression in endothelial cells, lymphocytes and leukocytes but not adipocytes, liver, kidney and heart, compared with $Lrp1^{f/f}$;$Tie2Cre^{-}$ (Cre-) mice. (**b**) Following bone marrow transplantation (BMT), Cre+ mice displayed the specific depletion of Lrp1 in endothelial cells and the recovery of Lrp1 mRNA levels in hematopoietic cells. n=3. *, $P<0.05$, compared to Cre- (**a**) or Cre-/BMT cells (**b**). (**c-d**) The confocal imaging analysis demonstrates the specific knockout of Lrp1 in liver sinusoidal endothelial cells (**c**) or epididymal fat endothelial cells (**d**). The staining for cross-sections of liver or epididymal fat tissues was performed with indicated antibodies of Lrp1 (8G1 antibody) and CD31 (an endothelial cell marker). Images presented are representative results of 4 sections per mouse in each group. Scale bars, 5 μm. (**e**) Body weight gain of Cre- and Cre+ mice was measured over time on high-fat (HF) diet or control chow (CC). Cre+ mice had significantly lower body weight compared to Cre- mice after 12 and 16 weeks of high-fat feeding. (**f**) The spleen and kidney tissue masses of Cre+ or Cre- mice were compared following 16 weeks of different diet feeding. Tissue masses were normalized by femur length (FL). n=4 for Cre+ mice and 5 for Cre- mice. *, $P<0.05$. Analysis was two-way ANOVA followed by Fisher's LSD multiple comparison test (for **a, b, e, f**).

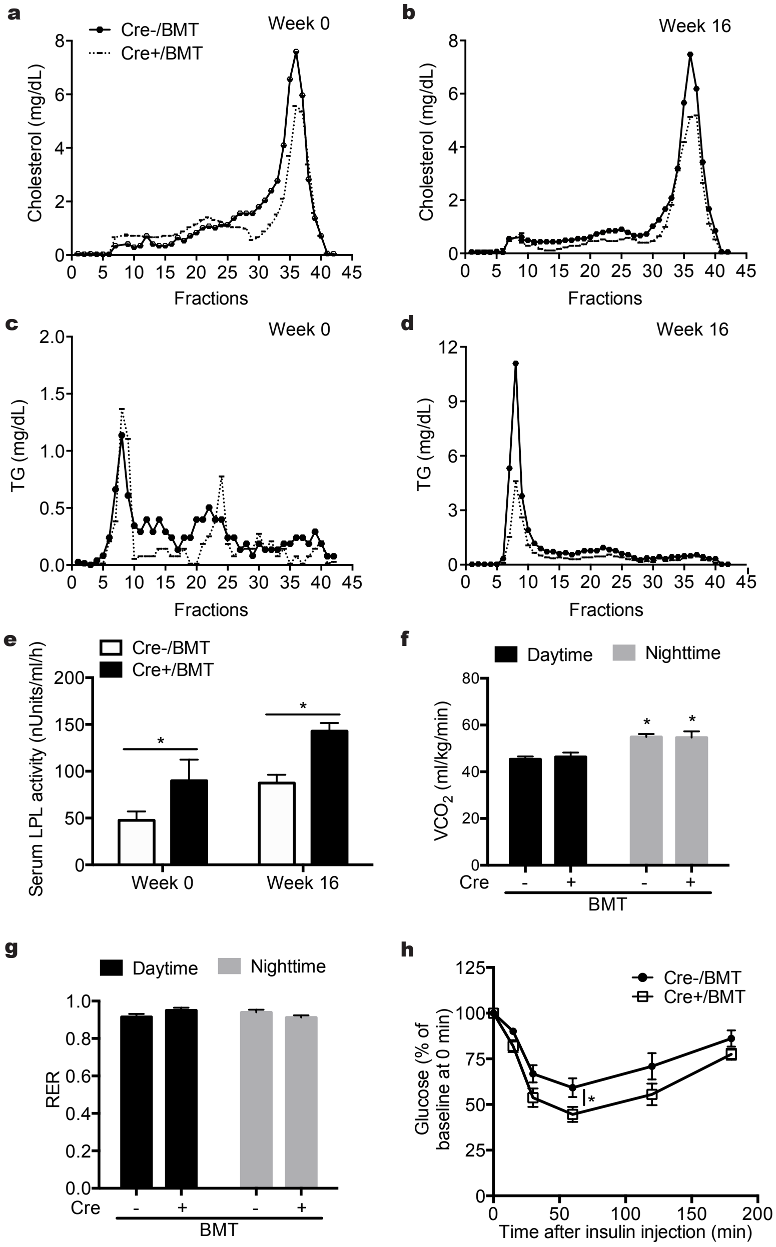

Supplementary Figure 2 Endothelial cell-specific Lrp1 depletion in mice results in improved metabolic responses. (**a-d**) Lipoprotein profiles were analyzed with FPLC in Cre+/BMT and Cre-/BMT mice before and after high-fat diet feeding for 16 weeks (Week 0 or 16, respectively). (**e**) Mouse serum LPL activity was analyzed before and after high-fat diet feeding for 16 weeks (Week 0 or 16, respectively). (**f-g**) VCO_2 (**f**) was measured and RER (respiratory exchange rate, **g**) was calculated in mice by metabolic cage studies before high-fat diet feeding. (**h**) Insulin tolerance tests were performed with Cre+/BMT and Cre-/BMT mice after high-fat diet feeding (Week 16). *, P<0.05. n=8 for Cre+/BMT mice and 6 for Cre-/BMT mice. Analysis was two-way ANOVA followed by Fisher's LSD multiple comparison test (for **e-h**).

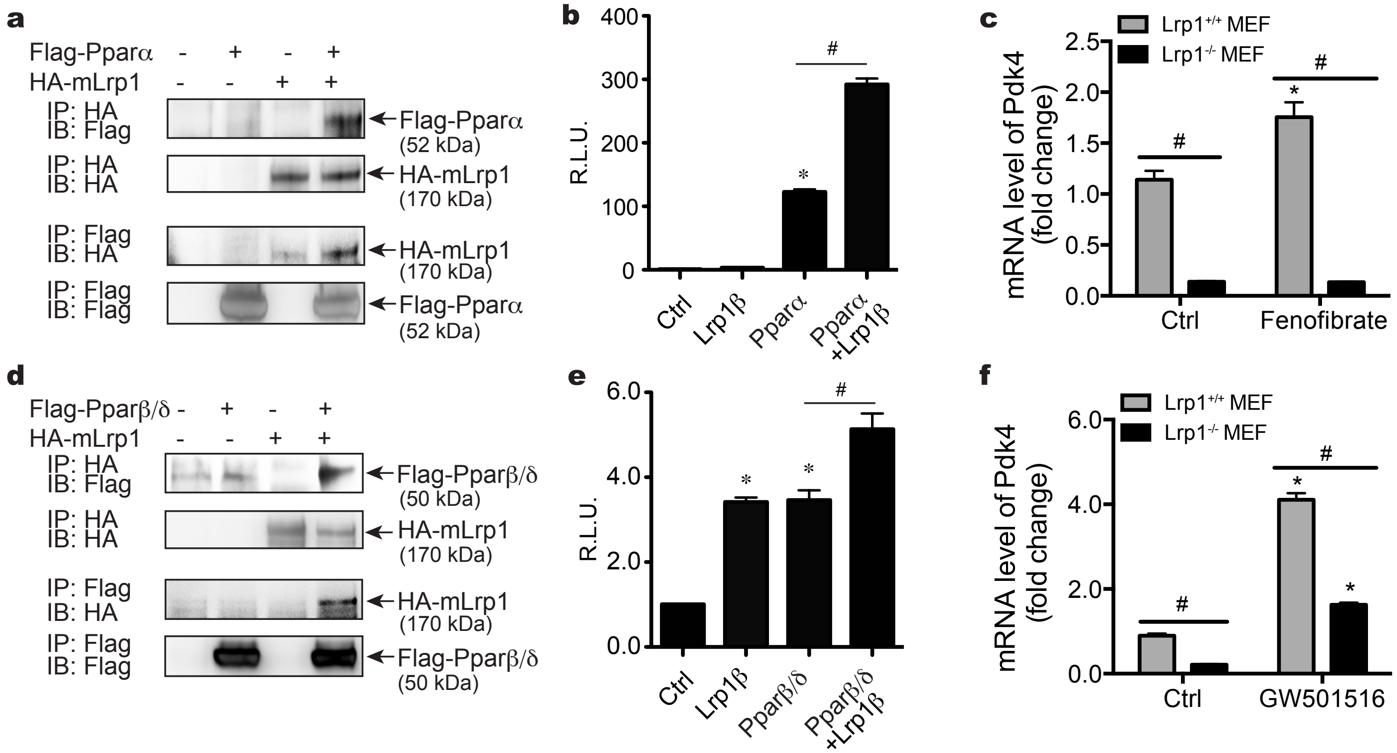

Supplementary Figure 3 Lrp1β binds to PPARα and PPARβ/δ and promotes their transcriptional activity. (**a, d**) Lysates of HEK 293 cells with over-expressed Flag-tagged PPARα (**a**) or PPARβ/δ (**d**) and HA-tagged mini-Lrp1 receptor (HA-mLrp1) were immunoprecipitated with an anti-Flag or anti-HA resin and blotted with an anti-HA or Flag antibody, respectively. (**b, e**) PPAR reporter assay was performed in HEK293 cells. Constructs of reporter gene PPRE-Luc, internal control renilla, Flag-PPARα (**b**) or PPARβ/δ (**e**), and Flag-Lrp1β were transfected into HEK293 cells. The firefly luciferase activity was then measured. (**c, f**) mRNA level of PDK4 was measured with real-time PCR assays. Lrp1 knockout mouse embryonic fibroblasts ($Lrp1^{-/-}$ MEFs) and wild-type ($Lrp1^{+/+}$ MEFs) were treated with 10 μM fenofibrate (an agonist of PPARα) or 100 nM GW501516 (an agonist of PPARβ/δ), and then mRNA was isolated. n=3. *, $P<0.05$, compared to same cells at control condition. #, $P<0.05$. Analysis was one-way ANOVA (for **b, e**), and two-way ANOVA followed by Fisher's LSD multiple comparison test (for **c, f**).

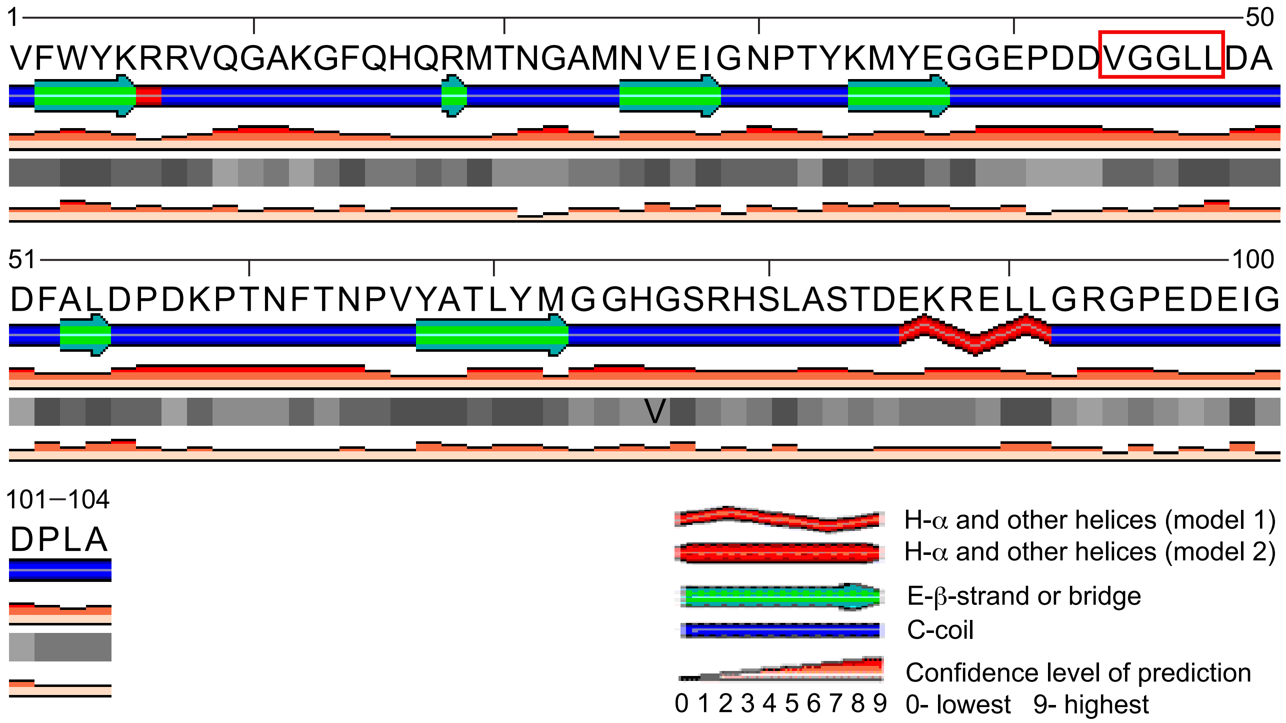

Supplementary Figure 4 The prediction of the secondary structure for the intracellular domain of LRP1β (NP_002323, a.a. 4441-4544), analyzed by the SABLE server prediction.

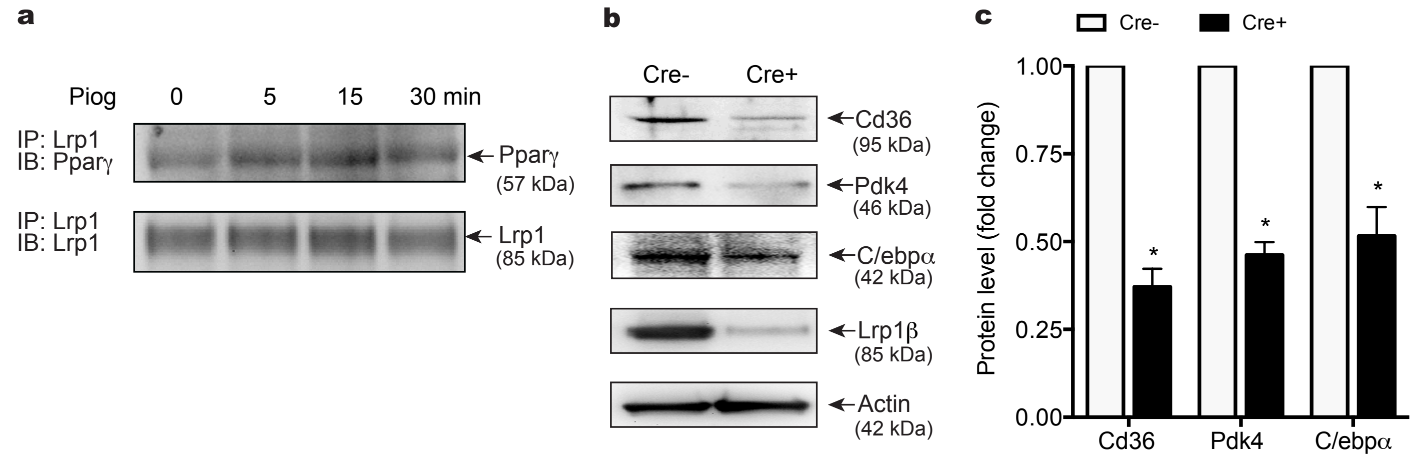

Supplementary Figure 5 Lrp1 interacts with PPARγ and regulates its target genes' expression. (**a**) Pioglitazone treatments increased the interaction of Lrp1 and PPARγ in endothelial cells. Lysates of primary mouse endothelial cells following treatments of pioglitazone at 10 μM for indicated time periods were immunoprecipitated with anti-Lrp1 C-terminal antibody and analyzed by Western blotting with an anti-PPARγ antibody. (**b-c**) Protein levels of CD36, PDK4 and C/EBPα were measured by Western blotting with isolated primary mouse endothelial cells from $Lrp1^{f/f}$;CAG-$CreER^{+}$ (Cre+) or $Lrp1^{f/f}$;CAG-$CreER^{-}$ (Cre-) mice. The band intensity of these proteins was normalized to actin and presented in (**c**). n=3. #, $P<0.05$, compared to Cre- ECs. Analysis was multiple unpaired Student's *t*-test (for **c**).

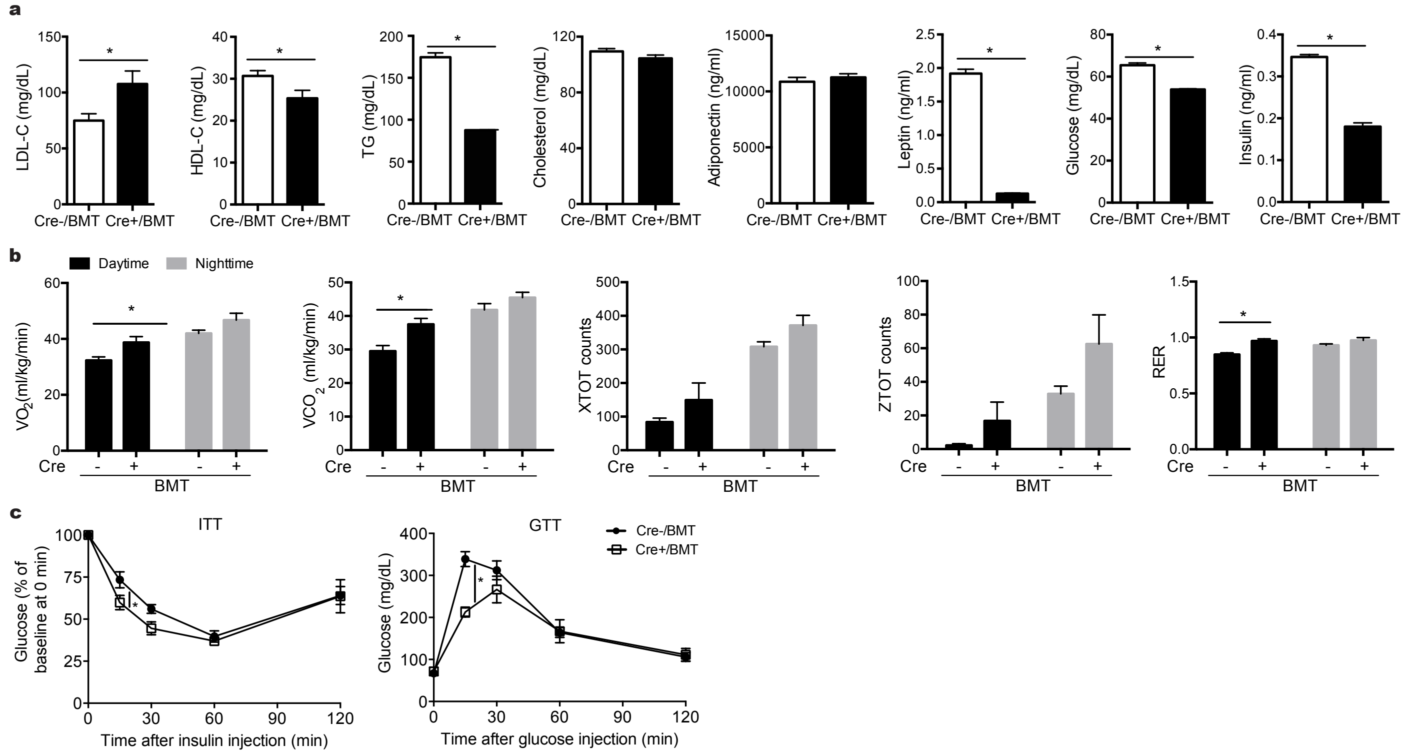

Supplementary Figure 6 Mice with endothelial Lrp1 depletion display improved metabolic responses following the treatment of rosiglitazone. (**a**) Metabolic parameters including plasma levels of LDL-cholesterol (LDL-C), HDL-cholesterol (HDL-C), triglyceride (TG), total cholesterol, adiponectin, leptin, fasting glucose and insulin were analyzed in Cre+/BMT and Cre-/BMT mice after rosiglitazone treatments for 3 weeks. (**b**) VO_2, VCO_2, locomotor activity in x-axis (XTOT) and z-axis (ZTOT), and RER (respiratory exchange rate) were measured in mice by metabolic cage studies after the treatment of rosiglitazone. (**c**) Insulin tolerance tests (ITT) and glucose tolerance tests (GTT) were performed with Cre+/BMT and Cre-/BMT mice after the treatment of rosiglitazone. *, $P<0.05$. n=4 for Cre+/BMT mice and 6 for Cre-/BMT mice. Analysis was unpaired Student's *t*-test (for **a**), and two-way ANOVA followed by Fisher's LSD multiple comparison test (for **b, c**).

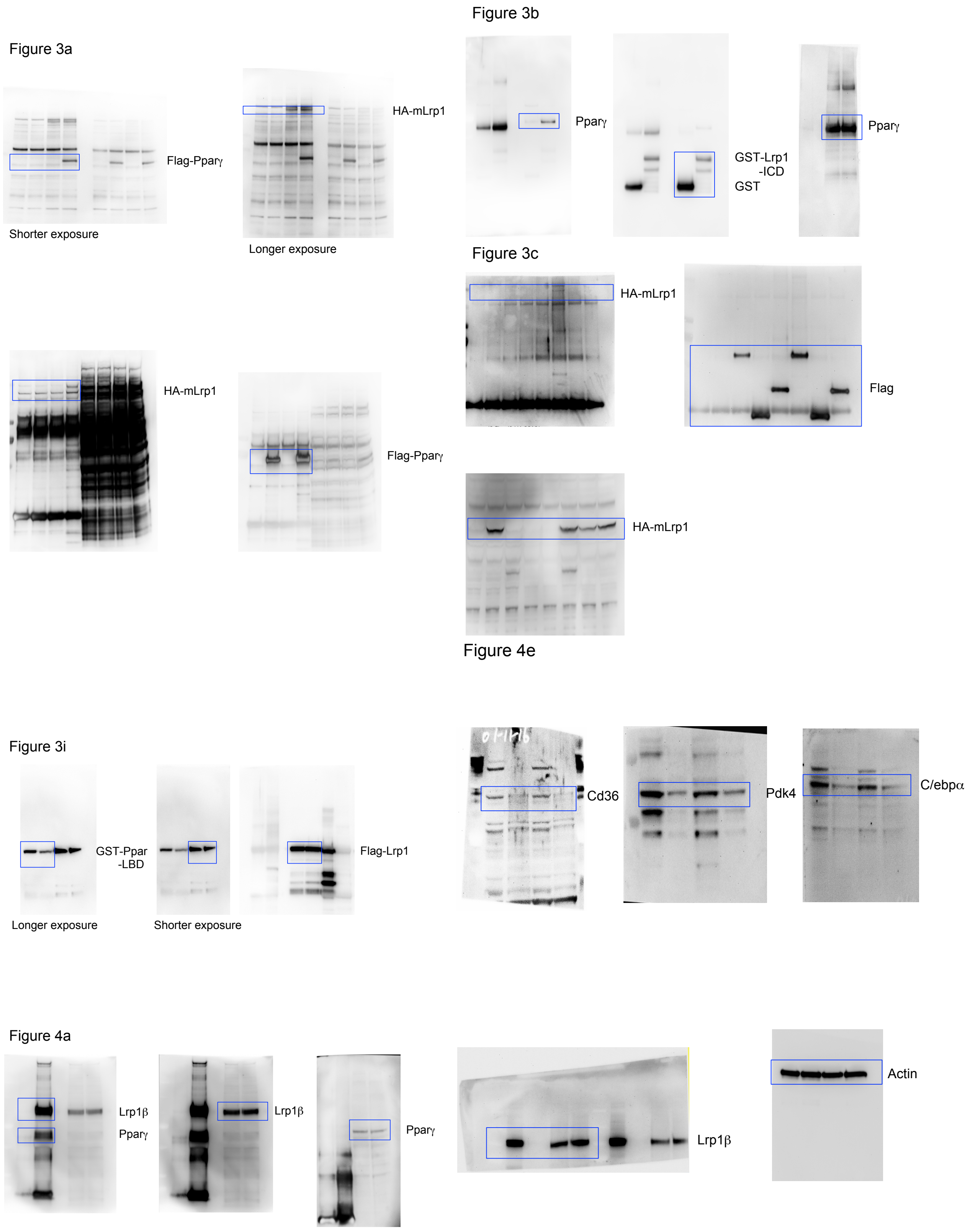

Supplementary Figure 7 Uncropped Western blotting images for figures.

Supplementary Table 1 Alignment of LRP1 coil sequence with nuclear receptor and co-activator helix motifs (adapted from the publication by Nolte RT et. al. [1]).

NR/NCoA coordinate motif	Amino acid sequence alignment
SRC-1, HD1	TSHK***LVQLL***TTT
SRC-1, HD2	RHKI***LHRLL***QEG
SRC-1, HD3	DHQL***LRYLL***DKD
NCoA-2, HD1	GQTK***LLQLL***TTK
NCoA-2, HD2	KHKI***LHRLL***QDS
NCoA-2, HD3	ENAL***LRYLL***DKD
P/CIP, HD1	GHKK***LLQLL***TCS
P/CIP, HD2	KHRI***LHKLL***QNG
P/CIP, HD3	NNAL***LRYLL***DRD
PPAR-g	LHPL***LQEIY***KDL
Thyroid receptor-α	FPPL***FLEVF***EDQ
RXR-α	IDTF***LMEML***EAP
RAR-γ	MPPL***IREML***ENP
Oestrogen receptor-α	LYDL***LLEML***DAH
Glucocorticoid receptor	FPEM***LAEII***TNQ
Progesterone receptor	FPEM***MSEVI***AAQ
LRP1, Coil	EPDD***VGGLL***DAD

References

1. Nolte, R.T., *et al*. Ligand binding and co-activator assembly of the peroxisome proliferator-activated receptor-gamma. *Nature* **395**, 137-143 (1998).
